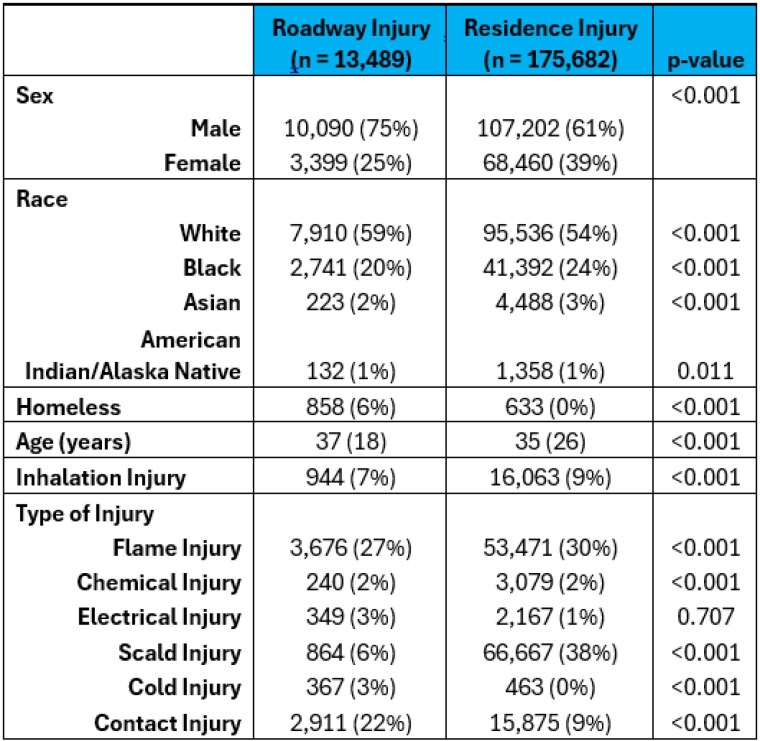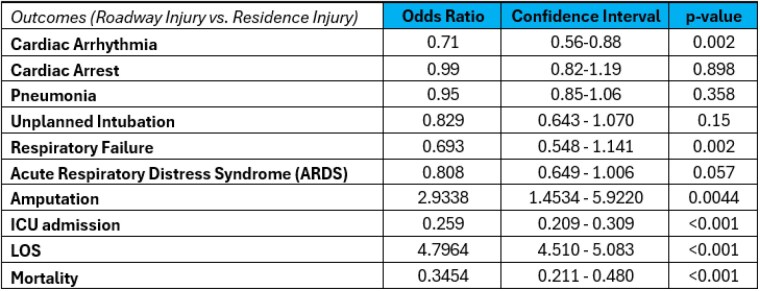# 583 Location Affects Outcomes: Private Residences vs. Roadways

**DOI:** 10.1093/jbcr/iraf019.212

**Published:** 2025-04-01

**Authors:** Tyler Murphy, Arman Fijany, Emily Swafford, Jordan Garcia, Punit Vyas, Robel Beyene, Stephen Gondek, Anne Wagner, Elizabeth Slater

**Affiliations:** Vanderbilt Burn Center; Vanderbilt University Medical Center; Vanderbilt Burn Center; Vanderbilt University Medical Center; Vanderbilt University Medical Center; Vanderbilt University Medical Center; Vanderbilt University Medical Center; Vanderbilt University Medical Center; Vanderbilt University Medical Center

## Abstract

**Introduction:**

The setting where a burn injury occurs impacts subsequent morbidity, outcomes, and clinical management. Understanding the differences in physical space could aid in the management and treatment protocols of these injuries. Albeit most burn injuries occur in private residences (PR), the recent increase in burn injuries occurring on roadways has never been reported on. We hypothesized that those who suffer from injury on a roadway will have increased mortality and associated comorbidities.

**Methods:**

The American Burn Association (ABA) Noncommercial Burn Research (NBR) Dataset for burn center admissions from 2012-2021 was queried for injury locations in PR vs. roadways. Within this retrospective cohort our primary outcome measured was mortality. Secondary outcomes investigated were ICU admission, hospital length of stay (LOS), cardiac arrhythmia, cardiac arrest, pneumonia, unplanned intubation, respiratory failure, and acute respiratory distress syndrome (ARDS). Multivariate regression with covariate adjustments for age, sex, percent total body surface area (TBSA) was performed for primary and secondary outcomes. To compare amputation rates, an odds ratio (OR) was calculated with the chi-square test.

**Results:**

A total of 13,489 (7%) burn injuries occurred on a roadway compared to 175,682 (93%) in a PR. Since 2012 there has been a linear increase in the ratio of roadway to PR injuries (r = 0.95). Burn injuries on roadways were significantly associated with older (mean 37 years [SD 18] vs. 35 years [26]), homeless (6% vs. 0%), males (75% vs. 61%) who suffer from cold (3% vs. 0%) or contact (22% vs. 9%) injury. In multivariable analysis, roadway injury was associated with increased risk of mortality (odds ratio [OR] 0.34; 95% confidence interval [CI] 0.2,0.5), ICU admission (OR 0.25; CI 0.2, 0.3), and extended LOS by five days (OR 4.80; CI 4.5, 5.1). These patients were also associated with greater risk of cardiac arrhythmia (OR 0.71; CI 0.6, 0.9), respiratory failure (OR 0.69; CI 0.5, 1.1), ARDS (OR 0.81; CI 0.6, 1.0), and amputations (OR 2.93; CI 1.5, 5.9).

**Conclusions:**

Our analysis found strong evidence for increasing burn admissions due to injuries occurring on roadways since 2012. These individuals have increased risk of mortality, ICU admission, and LOS. Additionally, these individuals have increased risks of complications including respiratory failure and amputations likely secondary to their mechanism and setting of injury. Limited by the usage of variables supplied by a national database in this study, future studies should investigate further on this increasingly present clinical scenario of roadway burn injury and further associate it with concurrent traumatic injury.

**Applicability of Research to Practice:**

As the first nationwide database study to describe the rising risk of roadway injury in the burn population, the findings within this abstract brings to light the importance of injury setting and the complications that come with roadway-based injury.

**Funding for the Study:**

N/A